# Montreal Cognitive Assessment scale in patients with Parkinson Disease with normal scores in the Mini-Mental State Examination

**DOI:** 10.1590/1980-57642018dn13-010008

**Published:** 2019

**Authors:** Krisly Arguedas Vásquez, Erick Miranda Valverde, Daniel Valerio Aguilar, Henri-Jacques Hernández Gabarain

**Affiliations:** 1 Geriatrician-Gerontologist Hospital Carlos Luis Valverde Vega, Caja Costarricense de Seguro Social, Alajuela, Costa Rica.; 2 Geriatrician-Gerontologist Memory Clinic, National Hospital of Geriatrics and Gerontology, Caja Costarricense de Seguro Social, San José, Costa Rica.; 3 Physician Neurologist Memory Clinic, National Hospital of Geriatrics and Gerontology, CAja Costarricense de Seguro Social, San José, Costa Rica.

**Keywords:** Parkinson’s disease, mild cognitive impairment, cognitive impairment, Montreal Cognitive Assessment, doença de Parkinson, comprometimento cognitivo leve, comprometimento cognitivo, Montreal Cognitive Assessment

## Abstract

Several screening tests have been used for cognitive evaluation in Parkinson’s disease (PD).

**Objective:**

To evaluate the usefulness of the Montreal Cognitive Assessment (MoCA) in patients with Parkinson’s disease and no cognitive impairment complaints.

**Methods:**

A total of 40 PD patients with no complaints of cognitive problems were included. Patients were selected using the Mini-Mental State Examination (MMSE) and the MoCA was then administered.

**Results:**

80% of patients exhibited Mild Cognitive Impairment (MCI) according to the MoCA. Statistically significant differences in visuospatial, attention and delayed recall functions were evident between the normal and abnormal MoCA groups.

**Conclusion:**

The study results suggest that MoCA may be a good screening test in patients with PD who do not present cognitive complaints.

## RESUMO

Vários testes de triagem foram utilizados para avaliação cognitiva na doença de Parkinson (DP).

**Objetivo:**

Avaliar a utilidade da Avaliação Cognitiva de Montreal (MoCA) em pacientes com doença de Parkinson sem queixa de comprometimento cognitivo.

**Métodos:**

Um total de 40 pacientes com TP sem queixas de problemas cognitivos foram admitidos e com o Estado de Exame do Estado Mental Mini (MEEM) foram selecionados e receberam o MoCA.

**Resultados:**

80% apresentaram dados de Comprometimento Cognitivo Leve (ICM) segundo o MoCA, sendo as funções visoespaciais, atenção e memória atrasada aquelas que apresentaram diferenças estatisticamente significantes entre os grupos MoCA normal e anormal.

**Conclusão:**

Este estudo sugere que o MoCA pode ser um bom teste de triagem em pacientes com DP que não apresentam queixas cognitivas.

Parkinson’s disease (PD) is the second most common neurodegenerative disease after Alzheimer’s disease (AD). As many as 20-30% of patients with Parkinson’s disease (PD) have cognitive deficits within the range of Mild Cognitive Impairment (MCI) at the time of diagnosis, a condition that is very important to detect since it is associated with an increased risk of developing dementia.^1^^,^ 2 The typical profile of cognitive impairment in PD involves impairment in executive functions, attention, visuospatial and subcortical memory functions, recall, language preservation, and praxis.^3^^,^ 4 Given this scenario, it is important to screen patients with PD because of the risk of developing dementia associated with PD (PDD).

Currently, several screening tests have been recommended for the evaluation of cognitive impairment in PD, such as the Scale for Outcomes of Parkinson’s Disease Cognition (SCOPA-COG), Mini-Mental Parkinson (MMP), Parkinson Neuropsychometric Dementia Assessment (PANDA), Parkinson Disease Dementia-Short Screen (PDD-Short Screen) and the Montreal Cognitive Assessment (MoCA).^5^ This last test was originally designed for the evaluation of mild cognitive impairment associated with AD, and evaluates memory, executive functions and verbal fluency among others, and can be applied in a short period of time. It has been used for the cognitive evaluation of the patients with PD to identify the presence of cognitive deficit when the MMSE score is normal. This test has a maximum value of 30 points and can be administered in 10 minutes. At least four studies have been conducted demonstrating that the MoCA in patients with PD is sensitive to small cognitive changes. The MoCA has shown a good test-retest effect, low inter-evaluator variability, sensitivity of 82% and specificity of 75% with respect to neuropsychological tests for detecting MCI and Dementia using a 26-point cut-off.^6^^,^ 7 MoCA has proven the most suitable instrument for screening Mild Cognitive Impairment in PD (PD-MCI) and PDD in clinical trials according to the Parkinson Study Group (PSG) Cognitive/Psychiatric Working Group.

The objective of this research was to evaluate the usefulness of the MoCA in the detection of cognitive impairment in PD patients without cognitive complaint and with normal MMSE score.

## METHODS

A total of 40 PD patients were selected from Neurology outpatient consultations at the National Hospital of Geriatrics and Gerontology. Disease diagnosis was according to the criteria of the United Kingdom Parkinson’s Disease Society Brain Bank Diagnostic Criteria for Parkinson’s Disease. Patients with disease duration <10 years, no cognitive complaints and an MMSE^8^^,^ 9 score ≥24 points were included. During a 12-month period, all patients underwent a complete anamnesis, a neuroimaging study consisting of Computed Axial Tomography scan, general biochemistry workup consisting of hemogram, renal function, electrolytes, renal and hepatic function tests, thyroid function, VDRL (Venereal disease research laboratory), levels of B12 vitamin and folic acid, in order to exclude other causes that might explain the cognitive deterioration. In addition, participants completed the Clock Drawing Test (scored according to Cacho et al.),^10^^,^ 11 Barthel’s Scale of Activities of Daily Living^12^ ( BADL), Lawton’s Scale of Instrumental Activities of Daily Living^12^ (IADL), the Yesavage Geriatric Depression Scale^13^ (GDS) 15-point version, the Hoehn and Yahr scale,^14^^,^ 15 as well as the MoCA test. Cases with a previous diagnosis of mild cognitive impairment, dementia, or psychiatric illness including depression, an advanced medical condition or severe sensory deficit were excluded.

In all cases, the different scales were applied by the same evaluator, trained in the use of all the tests, in order to standardize the evaluation. Basic sociodemographic information was also collected. The study was approved by the Local Scientific Ethics Committee (CLOBI) under number 03-2014.

Statistical analysis was performed using the statistical program Stata 13.0 SE (StataCorp. LP 2013). The presence of normal cognition was defined for scores ≥26 points on the MoCA and cognitive impairment as score <26, as determined for the general population. The comparison between the groups with and without cognitive impairment was performed using the Mann-Whitney U-Test.

## RESULTS

Among the total cases, females predominated (55%), while average age and formal education were 76.6±6.6 and 7.4±5.3 years, respectively. The mean duration of the disease was 5.5±3.2 years and 85% used Levodopa as part of their baseline therapy (Table 1).

**Table 1:** Clinical and epidemiological characteristics of patients with PD.

Variable		Total (n=40)	Percentage
Gender	Female	22	55 %
	Male	18	45 %
Age		76.6± 6 .6	
Marital status	Married	20	50 %
	Widowed	9	23 %
Education		7.4± 5 . 3	
Duration of disease		5.5±3.2	
Anti-Parkinson's treatment	Levodopa	34	85 %
	Dopaminergic agonist	5	12.5 %
	Amantadine	1	2.5 %
Hoehn and Yahr	Stage 1	15	37.5 %
	Stage 2	11	27.5 %
	Stage 3	8	20 %
	Stages 4 and 5	0	0%

In terms of level of functioning, 65% were at initial functional stages according to the Hoehn and Yahr scale, exhibiting slight repercussions on the BADL and the IADL scales, and had low risk of depression (Table 2).

**Table 2:** Cognitive and functional scale scores of PD patients.

Variable	Minimum	Maximum	Mean	Standard deviation
Basic activities of daily living (Barthel)	30	100	89.6	15.9
Instrumental activities of daily living (Lawton)	0	8	4.8	2.3
Geriatric depression scale (Yesavage)	0	11	2.3	2.6
MMSE	24	30	26.7	2.9
Clock Drawing Test	1	10	6.8	2.9
MoCA	11	30	20.7	4.8

MMSE: mini-mental state examination; MoCA: Montreal Cognitive Assessment.

Regarding cognitive aspects, the average score obtained on the MMSE was 26.7 and on the Clock Drawing Test was 6.8 points. The mean value on the MoCA was 20.7 points, proving abnormal (score <26) in 80% of the cases (Tables 2 and 3). For the different MoCA sub areas, statistically significant differences in visuospatial, attention and delayed recall functions were evident between the normal and abnormal MoCA groups (Table 3).

**Table 3:** MoCA Results for PD patients.

Cognitive subarea	MoCA < 26 points Mean and SD	MoCA ≥26 points Mean and SD	Z^a^ score	P-value
Total	32 (80 %)	8 (20 %)	NA	NA
Visuospatial/Executive	1.5±1.3	3.9±1.6	-3.3	0.001*
Naming	2.5±0.9	3.0±0.0	-1.8	0.075
Attention	3.3±1.3	5.5±0.8	-3.7	0.000*
Language	2.1±0.9	2.8±0.5	-1.9	0.057
Abstraction	1.8±0.5	2.0±0.0	-1.3	0.190
Delayed recall	1.3±1.3	3.4±1.4	-3.2	0.001*
Orientation	5.5±1.0	6.0±0.0	-1.7	0.074

MoCA: Montreal Cognitive Assessment.

a Mann-Whitney U-Test

* Significance 0.001.

## DISCUSSION

The main finding was that cognitive impairment in PD, defined as an MoCA score lower than 26 according to the recommended score, is a common finding in PD patients without cognitive impairment complaints. Despite normal MMSE scores in all cases, 80% of the patients had abnormal performance on the MoCA according to overall score. This finding indicated that the majority of patients with PD had some degree of cognitive deterioration on more extensive and sensitive neuropsychological tests such as the MoCA. On the other hand, the comparison of the different cognitive areas showed that visuospatial, attention and delayed recall functions were the most relevant, a result in line with other investigations.^16^^,^ 17 These findings suggest that initial cognitive impairment in PD patients occurs in a range of cognitive domains and highlights the importance of a thorough cognitive assessment using a simple but sensitive neuropsychological test. It is noteworthy that average scores on the Clock Drawing Test were lower than normal, correlating with alterations on the visuospatial/executive section of the MoCA; data to be considered as a way of compensating for the deficiencies of the MMSE in the evaluation of patients with PD.^17^^-^ 19 The present study corroborates the facts documented by many other investigations, showing that the MoCA can detect the presence of cognitive deterioration in patients with PD. This is of paramount importance because the presence of MCI represents a risk factor for subsequent development of dementia and instruments such as the MMSE lack the sensitivity and specificity to detect the condition.^20^

In conclusion, patients with PD can present cognitive impairment even when not perceived by the patients. The use of cognitive tests with greater sensitivity and specificity is required to detect these deficits and the MoCA represents a good alternative for this purpose.
